# Comparison of The Expression of miR-326 between Interferon beta Responders and Non-Responders in Relapsing-Remitting Multiple Sclerosis 

**DOI:** 10.22074/cellj.2020.6486

**Published:** 2019-09-08

**Authors:** Mahtab Fattahi, Nahid Eskandari, Fattah Sotoodehnejadnematalahi, Vahid Shaygannejad, Kazemi Mohammad

**Affiliations:** 1 Department of Biology, Science and Research Branch, Islamic Azad University, Tehran, Iran; 2 Department of Immunology, School of Medicine, Isfahan University of Medical Sciences, Isfahan, Iran; 3 Applied Physiology Research Centre, Isfahan Cardiovascular Research Institute, Isfahan University of Medical Sciences, Isfahan, Iran; 4 Department of Neurology, Isfahan Neurosciences Research Center, Isfahan University of Medical Sciences, Isfahan, Iran; 5 Department of Genetic and Molecular Biology, School of Medicine, Isfahan University of Medical Sciences, Isfahan, Iran

**Keywords:** Interferon-Beta, Lymphocyte, MicroRNA, Multiple Sclerosis

## Abstract

**Objective:**

Multiple sclerosis (MS) is an inflammatory disease resulting in demyelination of the central nervous system (CNS). T helper 17 (Th17) subset protects the human body against pathogens and induces neuroinflammation, which leads to neurodegeneration. MicroRNAs (miRNAs) are a specific class of small (~22 nt) non-coding RNAs that act as post-transcriptional regulators. The expression of the miR-326 is highly associated with the pathogenesis of MS disease in patients through the promotion of Th17 development. Recently, studies showed that disease-modifying therapies (DMTs) could balance the dysregulation of miRNAs in the immune cells of patients with relapsing-remitting MS (RRMS). Interferon-beta (IFN-β) has emerged as one of the most common drugs for the treatment of RR-MS patients. The purpose of this study was to evaluate the expression of the miR-326 in RRMS patients who were responders and non- responders to IFN-β treatment.

**Materials and Methods:**

In this cross-sectional study, a total of 70 patients (35 responders and 35 non-responders) were enrolled. We analyzed the expression of the miR-326 in peripheral blood mononuclear cells (PBMCs) of RRMS patients at least one year after the initiation of IFN-β therapy. Real-time polymerase chain reaction (RT-PCR) was applied to measure the expression of the miR-326.

**Results:**

The results showed no substantial change in the expression of the miR-326 between responders and non- responders concerning the treatment with IFN-β. Although the expression of the miR-326 was slightly reduced in IFN-β-responders compared with IFN-β-non-responders; however, the reduction of the miR-326 was not statistically significant.

**Conclusion:**

Overall, since IFN-β doesn’t normalize abnormal expression of miR-326, this might suggest that IFN-β affects Th17 development through epigenetic mechanisms other than miR-326 regulation.

## Introduction

 Multiple sclerosis (MS) is an inflammatory disease that leads to demyelination of the central nervous system (CNS). As the incidence of MS disease is rapidly increasing in recent decades, there is a serious need for the treatment, as well as the monitoring of the disease progression and evaluation of patients’ response to various therapies.

Recent investigations have shown that transplantation of human embryonic stem cell (hESC) is one of the promising therapeutic strategies in the field of cell-based treatment in MS (1, 2). Studies indicate hESCs play an essential role in the remyelination process and have the preventing roles in demyelination of neural cells (3).

Additionally, numerous biomarkers have been so far proposed such as transcription factors, cytokines, and microRNAs (miRNAs) for the monitoring of the disease progression, as well as the evaluation of drug efficacy in MS (4, 5). Although the etiology of MS disease is still opaque, it has been shown that proinflammatory Th1- and Th17-producing CD4^+^ T cells contribute to the pathogenesis of MS (6). Th17 subset protects the human body against pathogens and induces neuroinflammation, which leads to neurodegeneration (7, 8). 

MicroRNAs are a class of non-coding RNAs with a length of 22 nucleotides that act as post-transcriptional regulators. It has been implicated that miRNAs are involved in the proper function of the immune system and have a vital role in T cell differentiation. Also, the aberrant expression of miRNAs is associated with pathological conditions, such as autoimmune diseases (9). 

Some studies revealed that disease-modifying therapies (DMTs) could balance the dysregulation of miRNAs in the cells of the immune system in relapsing-remitting MS (RRMS) patients (10, 11). Studies have demonstrated that most of the miRNAs upregulated/downregulated during the disease course mediate the differentiation of Th17 cells. The expression of the miR-326 is linked to the pathogenesis of MS disease through the promotion of Th17 development (12).

To date, myriad studies have conducted on the alteration of miRNAs in response to disease-modifying treatments, indicating the importance of these types of RNAs in the monitoring of various disorders. Accordingly, some studies have focused on the changes in the profile expression of miRNAs in MS disease, and they showed that these molecules are altered during the course of disease and treatment (13). Several miRNAs, including miR-155 and miR-326, have been shown to act as regulators of the immune cell response. Thus, evaluating the expression of the miR-326 could be used as a biomarker for the assessment of the immune cell function in MS patients. Interferon-beta (IFN-ß) was the first disease-modifying drug used for the treatment of MS with long-lasting effect and well-tolerability (14). 

Hence, in this study, we examined whether the treatment of RRMS patients with recombinant IFN-ß influences the expression of the miR-326 in PBMCs of patients (15). To show whether RRMS patients are responder/ non-responder to IFN-ß therapy, the relapse rate and disability progression of patients during the disease course were assessed (16). Therefore, the present study aimed to evaluate the expression of the miR-326 in IFN-ß responder and IFN-ß-non-responder MS patients. 

## Materials and Methods

### Patients

A cross-sectional study was conducted to determine the level of the miR-326 expression in PBMCs of 70 RRMS patients from Isfahan city. The study enrolled 70 RRMS patients who were diagnosed as IFN-ß-responders (n=35) and IFN-ß-non-responders (n=35). The diagnosis of MS patients was made based on the McDonald’ criteria (17). All of RRMS patients were treated with IFN-ß for at least one year. Patients were classified based on the modified Rio score (MRS) (18). The modified Rio score is a simplified version of the Rio score, excluding the expanded disability status scale (EDSS) progression and modified items of the relapse rates and MRI activity. These scores are estimated after one year of IFN-ß therapy with the aim of characterizing MS patients that will have ongoing disease activity and become suboptimal responders in the following two years (19). MS patients are categorized as IFN-ß responders when the score of EDSS remains unchanged, and patients have no relapse during the follow-up period. Accordingly, non-responders are defined as RRMS patients whose EDSS is increased at least one point, and they experience at least one relapse during the follow-up period (Table 1) (20). The study was approved by the local Ethics Committee of Isfahan University of Medical Sciences (code. no. 296075), and all patients were given informed consents. Informed consent was obtained from all individual participated in our study. 

**Table 1:** Demographic and clinical characteristics of RRMS patients


Demographic data		Responders	Non-responders

Mean age (Y)		33.72 ± 8.19	35.44 ± 8.06
Sex			
	Female	n=30	n=29
	Male	n=5	n=6
EDSS score		0-5	0-5


RRMS; Relapsing-remitting multiple sclerosis and EDSS; Expandeddisability status scale.

### Peripheral blood mononuclear cells isolation

PBMCs were isolated from fresh heparinized venous blood by centrifugation over Ficoll-Hypaque. The isolated PBMCs were washed twice with phosphate- buffered saline (PBS, Sigma, Germany) at 1800 rpm for 10 minutes. The supernatant was removed, and the pellet was re-suspended into 2 ml of PBS. Trypan blue (Sigma, Germany) was used to determine the cell viability in the cell suspension. Then, PBMCs were rinsed with PBS at 800 g for 10 minutes. After removal of the supernatant, the cells were stored at -80°C until RNA isolation.

### RNA extraction and cDNA synthesis

Total RNA including microRNAs was extracted from PBMCs of RRMS patients using the RiboEx Kit (GeneAll, Korea) following the manufacturer’s instructions. The quantity and integrity of the isolated RNA were confirmed using a Nanodrop and agarose gel electrophoresis. For the analysis of the miR-326 expression, 2 µl of RNA (5 ng/ µl) was reverse transcribed into complementary DNA (cDNA) using miRCURY™ LNA™ miRNA RT Kit following the manufacturer’s (Exiqon, Denmark). 

###  Real-time polymerase chain reaction

The analysis of the microRNAexpression was performed using RealQ Plus Master Mix Green (Ampliqon, Denmark) and specific microRNA LNA™ PCR primer set (Exiqon, Denmark) on an ABI 7500 system. The fold change expression of microRNA was calculated using the 2-..ct method and expressed relative to the RNU48 expression level. Real-time polymerase chain reaction (PCR) was performed using a microRNA LNA™ PCR primer set (forward primer: CCTCTGGGCCCTTCCTCCAG) and the RealQplus 2xMasterMixGreenHigh ROX Kit containing the miScript Universal Primer (reverse primer). 

### Statistical analysis

The analysis of the miR-326 expression was carried out by the SPSS software version 22 (SPSS, Chicago, IL). The difference of the miR-326 expression between responders and non-responder MS patients to IFN-ß therapy was analyzed by Student t test, and the P<0.05 was statistically considered significant. 

## Results

As confirmed in previous studies the levels of miRNAs would be altered in MS patients considering whether they response to IFN-ß therapy (10, 11). 

### The expression of the miR-326 in responders and non- responders RRMS patients

**Fig.1:**
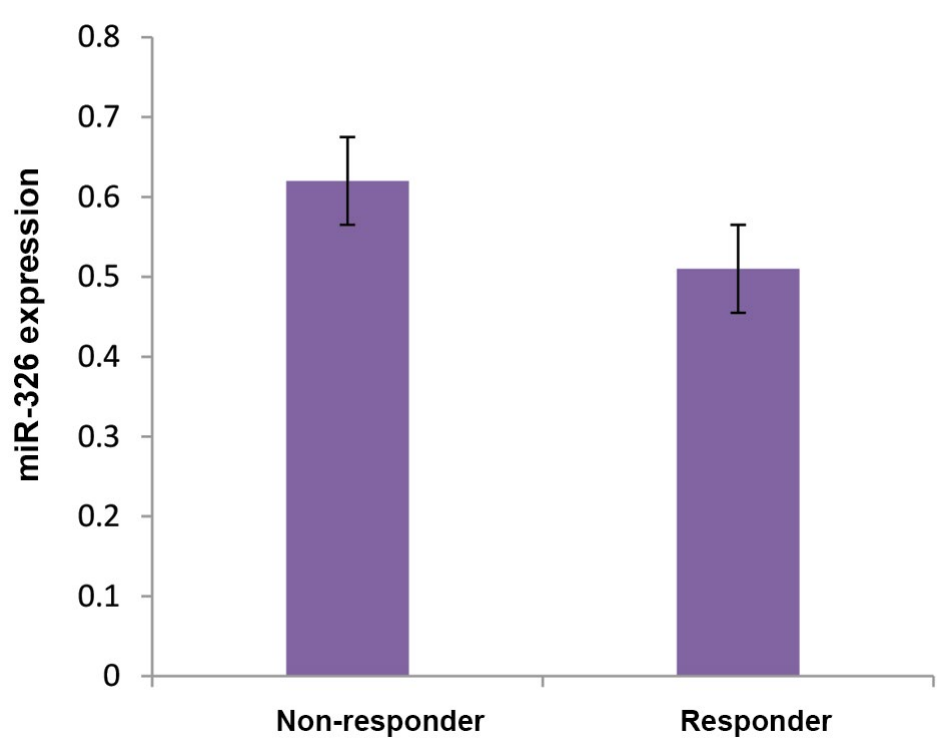
The RT-PCR analysis of miR-326 expression. The expression of the miR-326 was assessed in PBMCs of the responder and non-responder groups to IFN-ß. The results are presented as the ratio of miRNA to RNU48. The miR-326 was down-regulated in response to the treatment with IFN-ß. Although the expression of the miR-326 was higher in non- responder RRMS patients in comparison with responder RRMS patients, the difference is not statistically significant. Data are presented as mean ± SD. RT-PCR; Real time polymerase chain reaction, PBMCs; Peripheral blood mononuclear cells, IFN-ß; Interferon-beta, and RRMS; Relapsing- remitting multiple sclerosis.

To evaluate the miR-326 expression in response to IFN-ß therapy, the expression of the miR-326 at least one year after IFN-ß treatment was assessed. Furthermore, the expression of the miR-326 was compared between the responder and non-responder group. The real-time PCR analysis showed that the level of the miR-326 was lower in the responder group in comparison with the non- responder group; however, such a difference was not statistically significant (P= 0.7, P>0.05, Fig .1).

## Discussion

Several lines of evidence support that autoreactive T cells including Th1 and Th17 cells may mediate autoimmunity in the CNS, leading to axonal degeneration and demyelination (21-24). The aberrant expression of miRNAs is associated with pathological conditions, including autoimmune diseases. Studies have shown that some miRNAs are dysregulated in brain lesions and the blood samples of MS patients. The miR-326 has recently been identified as a crucial regulator of Th17 differentiation and found to promote CNS inflammation in EAE, known as a murine model of MS disease (12).

Moreover, dysregulation of the miR-326 has been reported in patients with MS that is associated with several pathological processes. Emerging evidence has demonstrated that various microRNAs are dysregulated in several types of immune cells in RR-MS and could be fine-tuned by DMTs. The degree of drug responsiveness to IFN-ß therapy varies among MS patients as some of them do not respond to therapy. However, there is no consensus on the methods to validate the degree of drug responsiveness in MS patients. Our objective was to evaluate an immunologically relevant miRNAs to classify RRMS patients as responders and non-responders. We focused on the profile expression of the miR-326 since it has been implicated in pro-inflammatory processes in MS pathology. IFNß therapy may regulate the expression of miRNAs and have benefits for MS patients; however, some patients do not respond to therapy (25-28). 

Factors contributing to the treatment failure in some patients are not fully understood. Lack of drug responsiveness in MS patients may stem from genetic, pharmacological, and pathological factors (29). The miR326 is epigenetically dysregulated in PBMCs and CD4+ T cells of RRMS patients (12). In the current study, we searched whether IFN-ß therapy affects the expression level of the miR-326 which has been previously implicated in the Th17-differentiation pathway. According to our findings, there was no significant difference considering the expression of the miR-326 between the responder and non-responder groups. Waschbisch et al. (10) consistently showed that the expression of the miR-326 did not significantly change between the untreated and IFN-ßtreated MS patients during at least three months. Likewise, Hecker et al. (11) demonstrated that IFN-ß therapy for at least one year did not normalize the aberrant expression of some miRNAs such as miR-326 which is differentially expressed in MS. 

## Conclusion

 Overall, the identification of miRNAs in the blood samples of responder and non-responder MS patients to IFN-ß therapy may provide useful biomarkers for the monitoring of the drug responsiveness and disease progression. Besides, the determination of the genetic profile of patients (pharmacogenetics) who are either responders or non-responders would shed light on our understanding about the role of genetics in drug responsiveness in MS patients. 
